# Attitudes Toward Consumption and Conservation of Tigers in China

**DOI:** 10.1371/journal.pone.0002544

**Published:** 2008-07-02

**Authors:** Brian Gratwicke, Judy Mills, Adam Dutton, Grace Gabriel, Barney Long, John Seidensticker, Belinda Wright, Wang You, Li Zhang

**Affiliations:** 1 Save The Tiger Fund, The National Fish and Wildlife Foundation, Washington D. C., United States of America; 2 Department of Zoology, Oxford University, Oxford, United Kingdom; 3 International Fund For Animal Welfare, Yarmouth Port, Massachusetts, United States of America; 4 World Wildlife Fund, Washington, D. C., United States of America; 5 Wildlife Protection Society of India, New Delhi, India; 6 Horizon key, Beijing, China; 7 College of Life Sciences, Beijing Normal University, Beijing, China; 8 National Zoological Park, Smithsonian Institution, Washington D. C., United States of America; University of Exeter, United Kingdom

## Abstract

A heated debate has recently emerged between tiger farmers and conservationists about the potential consequences of lifting the ban on trade in farmed tiger products in China. This debate has caused unfounded speculation about the extent of the potential market for tiger products. To fill this knowledge gap, we surveyed 1880 residents from a total of six Chinese cities to understand Urban Chinese tiger consumption behavior, knowledge of trade issues and attitudes towards tiger conservation. We found that 43% of respondents had consumed some product alleged to contain tiger parts. Within this user-group, 71% said that they preferred wild products over farmed ones. The two predominant products used were tiger bone plasters (38%) and tiger bone wine (6.4%). 88% of respondents knew that it was illegal to buy or sell tiger products, and 93% agreed that a ban in trade of tiger parts was necessary to conserve wild tigers. These results indicate that while Urban Chinese people are generally supportive of tiger conservation, there is a huge residual demand for tiger products that could resurge if the ban on trade in tiger parts is lifted in China. We suspect that the current supply of the market is predominantly met by fakes or substitutes branded as tiger medicines, but not listing tiger as an ingredient. We suggest that the Traditional Chinese Medicine community should consider re-branding these products as bone-healing medicines in order to reduce the residual demand for real tiger parts over the long-term. The lifting of the current ban on trade in farmed tiger parts may cause a surge in demand for wild tiger parts that consumers say are better. Because of the low input costs associated with poaching, wild-sourced parts would consistently undercut the prices of farmed tigers that could easily be laundered on a legal market. We therefore recommend that the Chinese authorities maintain the ban on trade in tiger parts, and work to improve the enforcement of the existing ban.

## Introduction

Wild tigers face unprecedented threats today, including reduction in habitat, depletion of prey and continued poaching. However, many tiger specialists agree that wild tigers face no greater threat than China's consideration of legalizing the trade in tiger products (Dinerstein, et al. 2007).

Recent reports have found that tiger-occupied tiger habitat has shrunk by as much as 41% in the last 10 years [1]. At the same time, Asia's 14 tiger-range countries [2] have experienced explosive growth in their human populations, which have doubled since 1965, reaching 3.2 billion in 2005 [3]. Economic growth in these countries also saw a doubling in average per-capita GDP between 1999 and 2006, leading to expanding markets fueled by increasingly wealthy consumers [4]. In addition to loss and fragmentation of tiger habitat due to clear cutting for timber, conversion to agriculture, mining and infrastructure, Asia's rural poor are penetrating further into forests to harvest key tiger prey species such as deer and wild pigs [5], [6]. Some tigers are killed as revenge for livestock depredation, but the primary direct threat to tigers is poaching by hunters to supply the lucrative black market in tiger skins and bones for ornamentation and health remedies respectively [7]. Recent press reports from Malaysia, Vietnam and China also point to the widespread occurrence of illegal markets for tiger meat.

Between 1990 and 1992, China recorded exporting 27 million units of tiger products [8]. In 1993, China banned its domestic trade in tiger bones and their derivatives to help implement the international tiger trade ban already in place under the Convention on International Trade in Endangered Species of Wild Fauna and Flora (CITES). China's 1993 ban closed down a significant legal industry in tiger bones and medicines made from tiger bones. At first, the ban was resisted by the traditional Chinese medicine (TCM) industry, but in time the traditional Chinese medicine community adapted, finding effective alternatives and embracing support of tiger conservation as both necessary and a social responsibility in keeping with its core premise of harmony with nature [9]. A 2005 TRAFFIC survey of over 600 TCM shops in China found that the supply tiger products had indeed dropped, and that fewer than 3% of shops claimed to stock tiger products compared with the 18% that did so in 1994 [10].

Despite these promising developments, poaching of tigers in India and Nepal, and trafficking in their skins and bones saw an increase in the early 2000s. Investigators from the Environmental Investigation Agency, the Wildlife Protection Society of India and other conservation organizations documented an expanding market for tiger skins for use in traditional robes used in the pan-Tibetan region of China, accounting for some of the resurgence in tiger poaching [11]. The rapid growth in demand from this market was linked to large seizures of tiger bone made in 2004 and 2005 in India and Nepal, marking a significant surge following a four-year lull in seizures. In 2005, researchers in India found that every tiger had been poached from Sariska Tiger Reserve, which had until then, been considered well protected and held 22 tigers in 2001, according to Project Tiger statistics. An India-wide tiger census that followed found that there were just 1,165 to 1,657 tigers remaining in India by 2007, about half of 2002 estimates. This drove down estimates of all remaining wild tigers to 3,600–4,600 [13].

In Indonesia too, evidence for a flourishing trade in tigers and tiger parts was documented during investigations by TRAFFIC in between 1999 and 2002 (Shepherd and Magnus, 2004). These surveys were repeated in 2006 and highlighted the continued prevalence of open tiger trade, and uncovered supply chains to China.

The situation was further complicated when businessmen who were already farming tigers in China petitioned China's central government to lift the 1993 ban on tiger trade to allow trade in products made from farmed tigers. Many tiger conservationists believe that re-igniting demand for tiger parts and products among China's 1.4 billion consumers would increase poaching of wild tigers because the demand for wild tiger parts would not be satisfied by these farmed tigers for two reasons; 1) medicine made from wild tigers are believed to be more effective according to the ancient tenets of traditional Chinese medicine, and 2) the demand for tiger products cannot be met from farms alone. Furthermore, a legal market of any kind would allow laundering of poached tiger products that would be virtually undetectable [14]. The 171 CITES member nations share these concerns and decided by consensus in June 2007 that “…tigers should not be bred for trade in their parts and derivatives” [15].

These developments have lead to polarized arguments about the potential effects on wild tigers of reopening trade in products from farmed tigers. There is speculation on both sides about latent demand for tiger products in China, consumer behavior, and preferences for wild versus farmed tiger parts [14]. In order to fill these knowledge gaps, Save The Tiger Fund commissioned a survey of the adult urban population in seven major Chinese cities to gather crucial baseline information on consumer behavior, demand and attitudes towards tigers and the use of their parts and derivatives. China's urban population was selected as the target survey population because 43% of China's 1.4 billion people live in urban areas, and their disposable income levels grew by 60% between 2000 and 2005 [16]. In 2005, their mean annual disposable incomes per capita ($1,490) were three times higher than those of their rural counterparts [16], who are less likely to be able to afford products made from tigers or get access to them.

## Methods

A total of 1,880 adult residents in seven Chinese cities were interviewed in April or May 2007 by Horizon key, an independent Chinese polling and research company. Demographic characteristics of respondents are listed in Appendix S1. The cities included: Kunming (n = 254); Guilin (n = 278); Harbin (n = 265); Chengdu (n = 269); Guangzhou (n = 273); Shanghai (n = 270); and Beijing (n = 271). Following methods in [17], a stratified survey design was chosen to randomly select neighborhood committees within each city. (A neighborhood committee is a formal organizational tier of local governance nested within a municipality [18]). Within each neighborhood committee, households were selected randomly. Once a sample household was identified, face-to-face interviews were conducted with randomly selected household members who: had lived in that location for at least 1 year; were 18 years or older; had not participated in any other surveys in the past six months; and were unrelated to, or friends with, any employee of Horizon or any other polling company. Respondents were presented with a gift to thank them for their participation after the 35–45 minute interview.

The original survey data set consisted of 315 potential responses that captured a comprehensive snapshot of respondent's tiger consumption behavior, attitudes towards tiger conservation and key demographic variables. We narrowed down the dataset to a few variables of key interest and analyzed the data provided by Horizon. The main focus of this analysis is to understand tiger consumption behavior, the demographics of tiger consumption, knowledge about tiger trade issues and laws, and attitudes towards wild tiger conservation. Straightforward tallies of attitudes and consumption rates were made, with no weighting of data from different sized cities. For demographic variables including age, sex, education and income levels, null hypotheses of ‘no difference’ were tested using SPSS 14.0. After testing for assumptions of normality, means were statistically compared using one-way ANOVAs, and homogeneous subsets were determined using post-hoc Tukey HSD tests. This paper summarizes our main findings.

## Results

### Consumption Patterns

**Figure 1:**
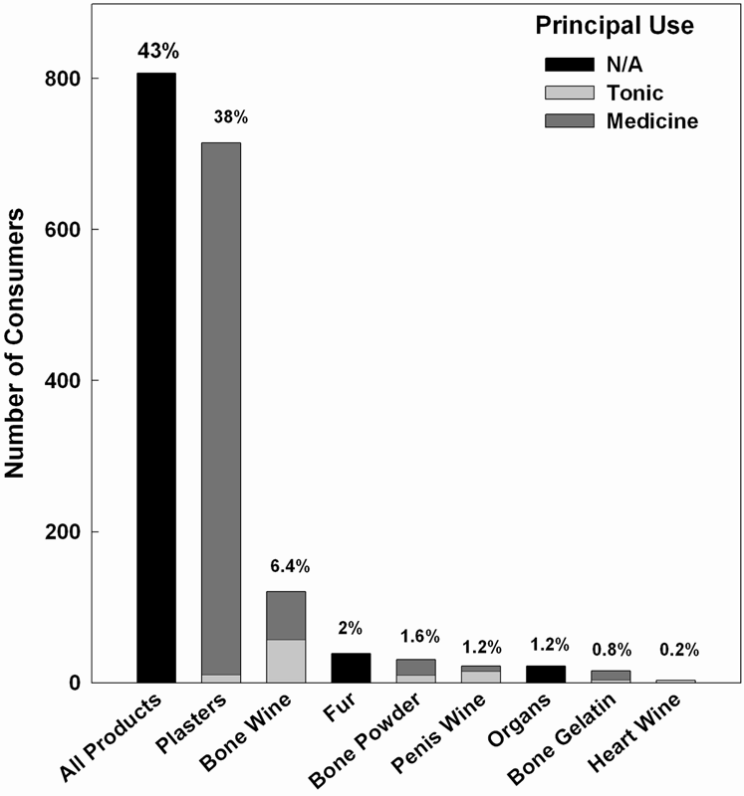
Survey of tiger product consumption in Chinese urban areas. Where applicable, respondents were asked to specify if the product was primarily used as a medicine, or as a health tonic. In this study we define medicine as a substance used to cure an illness, while a tonic is a substance used to primarily to promote general health and well being.

43% of all respondents had used some product thought to contain tiger derivatives (Figure 1), and 90% of these consumers stated that they had used tiger products since 1993, which is when China banned the sale of tiger bone and its derivatives. There was no way to verify whether consumers were using products that actually contained tiger derivatives, but 85% of consumers who had used tiger products admitted that they did not know whether the product they used was fake. 3% of these consumers believed that the product they were using was fake.

Tiger bone plasters, applied externally for aches and pains, were by far the most popular product, used by 38% of respondents (Figure 1). (Plasters are externally applied poultices containing a concoction of aromatic herbs and, sometimes contain animal derivatives such as tiger bone.) Of the respondents who had used tiger-bone plasters 60% had used the product in the last two years.

The only other alleged tiger product consumed by a significant number of respondents was tiger bone wine, which 6.4% of respondents claimed to have used. Of these, 52% said they consumed tiger bone wine in the past two years. Tiger bone wine was used equally as a medicine and as a health tonic. (In this study medicinal use was defined as used to cure an illness, while a tonic use was primarily to promote general health and well-being.) Both tiger bone plasters and tiger-bone wine were principally used to treat bone and joint-related conditions, such as arthritis and rheumatism, but tiger-bone wine was also taken as a tonic to increase sexual capacity (Table 1).

**Table 1:** The top 5 reasons cited for using tiger bone products.

	Tiger bone plasters	Frequency % (N = 715)	Tiger bone wine	Frequency % (N = 121)
**1**	To treat traumatic injury	74	To cure rheumatism	29
**2**	To cure rheumatism	57	To improve sexual capacity	23
**3**	To replenish calcium	38	To treat traumatic injury	20
**4**	Anti-inflammation	23	To replenish calcium	19
**5**	To treat hyperosteogeny*	9	To treat hyperosteogeny*	11

Respondents were asked to select the main reason(s) for use from a list of 10 known reasons; hence cumulative totals may exceed 100%.

* hyperosteogeny is a TCM term referring to osteoporosis or fragile bones.

**Figure 2:**
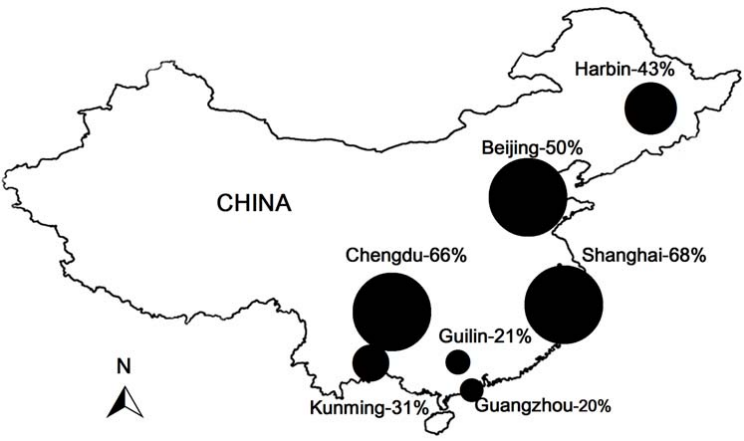
The prevalence of tiger consumption by residents of 7 Chinese cities. Prevalence of consumption was significantly different between cities (One way ANOVA, 6df, p<0.001). A post-hoc Tukey HSD test split the cities into the following homogeneous subsets a) Shanghai and Chengdu, b) Harbin and Beijing, c) Kunming, Guilin and Guangzhou.

Older people were significantly more likely (F_2,1879_ = 55.41 p<0. 001) to be consumers of tiger products than younger people, and women were more likely to use tiger-bone plasters than men (F_1,1879_ = 7.21- p<0.007) (Table 2). There was no significant difference in the likelihood of consumption of tiger-bone plasters between income groups ((F_2,1769_ = 0.42, p<0.65), but consumption of tiger-bone wine was only prevalent among wealthier consumers (F_2,1769_ = 0.24, p<0.02). Tiger consumption prevalence also varied significantly ((F_6,1879_ = 52.12, p<0. 001) depending on the city, with Chengdu and Shanghai being the consumption hotspots (Figure 2).

**Table 2:** The demographics of tiger consumption: Summary results of one-way ANOVAs testing null hypotheses about tiger consumption habits.

Null Hypothesis		F	P	Trend from post-hoc Tukey test
All age groups (3) are equally likely to consume:	All tiger products	F_2,1879_ = 55.41	**<0.001**	Younger people consume less
	Tiger plasters	F_2,1879_ = 62.05	**<0.001**	Younger people consume less
	Tiger bone wine	F_2,1879_ = 10.23	**<0.001**	People older than 45 are twice as likely to consume compared to younger age groups
Both genders (2) are equally likely to consume:	All tiger products	F_1,1879_ = 7.21	**0.007**	NS Accept null hypothesis
	Tiger plasters	F_1,1879_ = 9.84	**0.002**	Females consume more than males
	Tiger bone wine	F_1,1879_ = 2.84	**0.092**	NS Accept null hypothesis
People from all education levels (3) are equally likely to consume:	All tiger products	F_2,1868_ = 9.15	**<0.001**	People educated at the university-level consume less than those who have only been educated to the level of junior or senior school
	Tiger plasters	F_2,1868_ = 14.29	**<0.001**	People educated at the university-level are half as likely to be users of tiger plasters as those who have only been educated to the level of junior or senior school
	Tiger bone wine	F_2,1868_ = 0.70	**0.495**	NS Accept null hypothesis
People from all household income levels (3) are equally likely to consume:	All tiger products	F_2,1769_ = 2.26	**0.104**	NS Accept null hypothesis
	Tiger plasters	F_2,1769_ = 0.42	**0.652**	NS Accept null hypothesis
	Tiger bone wine	F_2,1769_ = 3.91	**0.02**	People with household incomes exceeding 4000 RMB are twice as likely to consume tiger bone wine as those whose household income is 2000 RMB or less
People from all cities (7) are equally likely to consume:	All tiger products	F_6,1879_ = 52.12	**<0.001**	Consumption highest in Chengdu and Shanghai, followed by Beijing and Harbin, Kunming, Guilin and Guanzhou consume the least (see Fig 2)
	Tiger plasters	F_6,1879_ = 71.23	**<0.001**	Consumption highest in Chengdu and Shanghai, followed by Beijing and Harbin, Kunming, Guilin and Guanzhou consume the least
	Tiger bone wine	F_6,1879_ = 1.80	**0.095**	NS Accept null hypothesis

A strong majority (71%) of consumers said they preferred to use tiger products from wild tigers over captive-bred tigers, while 7.6% said they preferred to use products from captive-bred tigers. This result was mirrored by the results to a question posed to all respondents “Which is more valuable [as a medicine], wild or farmed tigers?” In answer, 78% of respondents said that wild tigers were more valuable than farmed ones, and just 2% claimed that farmed tigers were more valuable. When questioned about substitutes, 54% of consumers said they were willing to use tiger-bone substitutes, while 30% said they were not.

### Attitudes and Knowledge

Most respondents were supportive of tiger conservation. 96% of respondents thought it was important to protect wild tigers, and 60% understood that restricting trafficking and regulating tiger trade were important actions that the government should undertake to save wild tigers (Table 3). At the same time, the status of tigers in the wild was poorly understood. About a third (32%) of respondents knew that there were fewer than 5,000 wild tigers, and only 10% knew that there were fewer than 50 wild tigers left in China (Table 3).

**Table 3:** Attitudes and knowledge of tiger conservation issues in Chinese cities (N = 1880, % rounded to nearest whole number).

Question	Response	%
**Do you think it is important to protect wild tigers?**	Very important	62
	Somewhat important	34
	Not very important	2
	Not important at all	0
	Refuse to answer/Don't know	2
**Which one do you think the most important work for Chinese government to conserve wild tigers?**	Improve protection of tiger habitat	27
	Enforce laws restricting tiger trafficking	33
	Improve supervision of the tiger trade	13
	Improve protection of tiger prey	8
	Improve education about tiger conservation	15
	Refuse to answer/Don't know	4
**Do you know how many tigers are left in the wild?**	Fewer than 5,000	32
	5,000–10,000	19
	10,000–50,000	6
	50,000+	2
	Don't know	40
**Do you know how many wild tigers there are in China?**	Less than 50	10
	50–100	19
	100–200	15
	more than 200	18
	Don't know	37
	Refuse to answer	1

With regard to laws related to tiger trade, 80% of respondents had not specifically heard of the 1993 state circular banning trade in tiger bone and rhino horn. However, only 12% thought that it was legal to sell tiger products (Table 4). Most respondents felt it was important to protect tigers and that enforcing laws regulating trade were needed to protect wild tigers (Table 4). Nearly all respondents (93%) agreed that the government should continue to ban the trade in wild tiger parts with 58% agreeing strongly and 35% agreeing somewhat. There was no significant relationship between a respondent's tiger consumption behavior and his/her level of support of the government trade ban (one-way ANOVA, F_5,1879_ = 2.1, p<0.06), but there was a trend, with the people most strongly disagreeing with the trade ban being more likely to consume tiger products. 53% of the 13 people strongly disagreeing with the trade ban were consumers; 35% of the 42 people somewhat disagreeing were consumers; 47% of the 664 people somewhat agreeing with the ban were consumers, but only 40% of the 1089 people agreeing strongly were consumers. The remaining 72 people either did not know or refused to answer.

**Table 4:** Attitudes and knowledge of tiger trade issues in Chinese cities (N = 1880, % rounded to nearest whole number).

Question	Response	%
**Which activities are legal in China?** *	Making donations to tiger farms and zoos with tigers	37
	Selling products labeled as tiger parts	12
	Buying or selling tiger antiques	8
	Domestic and international trade in tiger parts and products	8
	Purchasing tiger parts or products as an individual	4
	All above are illegal	35
	Don't know	14
	Refuse to answer	1
**Do you agree with the government prohibition on trade in tiger products?**	Disagree strongly	1
	Disagree somewhat	2
	Agree somewhat	35
	Agree strongly	58
	Don't know/Refuse to answer	4
**Which statements do you agree with?** *	Use of tiger products will cause extinction of wild tigers	60
	Use of tiger products is bad for the planet	36
	Use of tiger products is bad for China's image	26
	Use of tiger products is part of my Chinese heritage	11
	Use of tiger products is essential for my health	11
	Use of tiger products is old fashioned	6
	Use of tiger products is status symbol	6

* Applicants could select multiple responses, thus totals may exceed 100%.

## Discussion

### General Consumption Patterns

A total of 43% of respondents (807) said they had used a product claiming to contain tiger parts. 93% of these consumers had last consumed the alleged tiger product after the 1993 tiger trade ban went into effect. On this occasion no special technique was used to encourage an honest answer to these sensitive questions. It is therefore reasonable to surmise that the 40% admitting to carrying out an illegal activity is likely to be an underestimate of the total. Within the group of self-described consumers, 71% expressed a preference for wild tiger products.

In the context of this preference for products from wild tigers, it should be noted that the sale of products from the bones of a single wild caught tiger can be in the range of US$1,250–3,750 per kilogram, with an average of 20 kg of bones per tiger [10]. Considering the average per capita GDP in tiger-range countries is US$1,878[4] this provides ample incentive for poachers and smugglers to continue to catch and trade wild-caught tigers. Moreover, it is worth noting that the cost of raising a tiger in captivity is conservatively estimated to be US$4,000 [19] which has two significant consequences: 1) that it is always going to be more cost effective to poach wild tigers than to breed and raise them in captivity, and 2) that the average person in tiger-range countries cannot afford to raise tigers whereas they can afford the minimal costs that it takes to poach a wild tiger. In addition to this, without very strong enforcement and monitoring, the added economic benefit of laundering wild caught tigers through legal farming operations will always remain high enough for it to remain a threat to wild tiger populations.

### Tiger-Bone Plasters

The majority of tiger product consumers (88%) admitted having used tiger-bone plasters, and 60% of these said they used plasters in the past two years. People from all income groups used tiger-bone plasters, with the highest demand among older consumers and women. This is probably because older people tend to suffer from bone degeneration and arthritis and post-menopausal women are known to have higher incidences of rheumatoid arthritis and osteoporosis [20], which are primary ailments for which tiger-bone plasters are used.

It is common to find the tiger's image on plasters but the plasters do not list tiger bone as an ingredient, because that would be illegal. One study found that out of seven brands of plasters tested, none contained even traces of tiger bone [21]. In a 2005–2006 survey of 518 traditional medicine stores in China, no plasters listing tigers as an ingredient were found [10]. Therefore, one can probably assume that the bulk of plasters consumed by survey respondents did not contain tiger bone. It is interesting to note that despite the likely prevalence of fake products in the market, only 3% of the consumers believed that their products they purchased were fakes. Another 12% believed the products were real, while 85% were unsure whether the products used actually contained tiger ingredients. Since such a high percentage of people did not know whether or not tiger-bone plaster contained tiger bone ingredients or not, this may be an opportunity to engage the TCM industry to re-brand these plasters as bone-healing plasters, rather than tiger-bone plasters. This could relieve people's reservations about the legality of the product in question.

### Tiger-Bone Wine

Only 6.4% of survey respondents claimed to have consumed tiger bone wine. Unlike tiger-bone plasters, tiger-bone wine was used equally as a medicine and a tonic. The primary reasons for use of tiger-bone wine were for bone-related conditions and to ‘improve sexual capacity’. Consumers of tiger bone wine were primarily from wealthier income brackets possibly due to the high costs of the product which range from US$63 to US$124 for a 500 ml bottle, depending on how long the bones have been steeped in alcohol [22].

Some tiger farms in China sell “bone protecting wine” in tiger shaped bottles, which are touted by staff as containing authentic tiger bone. The manufacturers use a name that sounds like the word “tiger” but is written differently [22]. Labels sometimes list *Panthera leo*, the Latin name for lion, as an ingredient. Sale of products made with lion bone are not banned in China. Tests of some of this wine proved inconclusive because the DNA was too degraded to determine whether bones from cats of any kind were used [10].

### Conclusions

One of the most striking consumption patterns documented in this survey is that 43% of respondents said they had used a product claiming to contain tiger parts and most had done so during a time when the sale of any products containing tiger bone was illegal in China. Within this group of self-described consumers, 71% expressed a preference for wild tiger products, representing a huge potential market for wild-sourced tiger bone products if the sale of products from farmed tigers were legalized in China. Given that wild and farmed tiger products are indistinguishable, products from wild tigers could easily be “laundered” into a legal market, and vice-versa to satisfy either preference. This laundering opportunity coupled with the low overhead costs for “producing” a poached tiger, which can be less than US$20 in some range states, would very likely pose a significant incentive to poach wild tigers.

Critically, this study suggests that the potential market for tiger products in Chinese urban areas is enormous with 43% of Chinese urban adults over the age of eighteen representing a potential market of 157 million people [3]. If China were to legalize the trade in tiger parts, it is unlikely that supplies from a captive population of around 5,000 tigers could effectively flood a market of this size such that demand for wild caught tigers would be diminished. Furthermore, the clear preference for products from wild caught tigers shows that even if the demand for tiger products could be met from farmed tigers, a demand for wild caught tigers would remain. This is a critical point because the opening up of a legal trade would make it significantly more difficult to police the illegal trade as wild caught tigers and their products could be laundered through legal establishments. The results of this survey show that there remains a large trade in tiger products in China (whether they are genuine or not is for this argument irrelevant) despite this trade being illegal under the government's total ban. A total ban is a fairly simple regulatory mechanism to enforce, but given the high levels of tiger poaching in range-countries, apparently for Chinese markets it is clear that improvements need to be made. There is no reason to expect that even more complex legislation allowing markets for farmed tigers could stop indistinguishable wild-sourced tiger parts from entering the legal trade. Therefore the lifting of any ban on trade in tiger parts should not be considered and enforcement of the existing bans in tiger-range and consuming countries should be improved to a level where all illegal trade is stopped. While the number of respondents claiming to have used tiger products (43%) shows a significant latent market for real tiger products – from legal and/or illegal sources – it is heartening to note that nearly all (93%) support China's tiger trade ban for the sake of protecting wild tigers and China's international image.

## Supporting Information

Appendix S1 Demographic characteristics of respondents
(0.02 MB DOC)
