# Supplementary material for: Attitudes Toward Consumption and Conservation of Tigers in China
Source: PLoS One. 2008 Jul 2;3(7):e2544. doi: 10.1371/journal.pone.0002544 (PMC2435601; doi:10.1371/journal.pone.0002544)
Supplement: Appendix S1 — Demographic characteristics of respondents (0.02 MB DOC) [file pone.0002544.s001.doc]

**APPENDIX S1:** Demographic characteristics of 1880 respondents in Chinese cities. Responses in each category may not = 100% because ‘don’t know’ and ‘refuse to answer’ were not included.

| **Demographic Category** | **Variable** | **% respondents** |
| --- | --- | --- |
| Gender | Male | 40 |
|  | Female | 60 |
| Age | 18-30 | 26 |
|  | 31-45 | 35 |
|  | 46+ | 39 |
| Education | Junior School | 31 |
|  | Senior School | 41 |
|  | University | 27 |
| Monthly household income (RMB) | >2001 | 32 |
|  | 2001-4000 | 40 |
|  | 4001+ | 22 |
